# Validation of Swab Sampling and SYBR Green-Based Real-Time PCR for the Diagnosis of Cutaneous Leishmaniasis in French Guiana

**DOI:** 10.1128/JCM.02218-20

**Published:** 2021-01-21

**Authors:** Romain Blaizot, Stéphane Simon, Marine Ginouves, Ghislaine Prévot, Denis Blanchet, Christophe Ravel, Pierre Couppie, Magalie Demar, Cécile Nabet

**Affiliations:** a Department of Dermatology, Andrée Rosemon Hospital, Cayenne, French Guiana; b Tropical Biome and Immunophysiopathology (TBIP), Université de Lille, CNRS, INSERM, Institut Pasteur de Lille, U1019-UMR9017-CIIL-Centre d'Infection et d’Immunité de Lille, Centre Hospitalier de Cayenne, Université de Guyane, Cayenne, French Guiana; c National Reference Centre for Cutaneous Leishmaniasis (associate laboratory), Cayenne, French Guiana; d Laboratory of Parasitology-Mycology, Andrée Rosemon Hospital, Cayenne, French Guiana; e University of Montpellier, National Reference Centre for Leishmaniasis, University Hospital Centre of Montpellier, MiVEGEC, Montpellier, France; f Sorbonne Université, INSERM, Institut Pierre-Louis d’Epidémiologie et de Santé Publique, AP-HP, Groupe Hospitalier Pitié-Salpêtrière, Service de Parasitologie-Mycologie, Paris, France; Mayo Clinic

**Keywords:** cutaneous leishmaniasis, diagnostic test, PCR, neglected tropical disease

## Abstract

Recent studies have highlighted the interest in noninvasive sampling procedures coupled with real-time PCR methods for the detection of *Leishmania* species in South America. In French Guiana, the sampling method still relied on skin biopsies. Noninvasive protocols should be tested on a large annual cohort to improve routine laboratory diagnosis of cutaneous leishmaniasis. Therefore, we evaluated the performance of a new *Leishmania* detection and species identification protocol involving cotton swabs and SYBR green-based real-time PCR of the Hsp70 gene, coupled with Sanger sequencing. Between May 2017 and May 2018, 145 patients with ulcerated lesions compatible with cutaneous leishmaniasis were included in the study at the Cayenne Hospital and its remote health centers. Each patient underwent scrapings for a smear, skin biopsies for parasite culture and PCR-restriction fragment length polymorphism (RFLP) (RNA polymerase II), and sampling with a cotton swab for SYBR green-based PCR. The most accurate diagnostic test was the SYBR green-based PCR on swab samples, showing 98% sensitivity. The mean PCR cycle threshold (*C_T_*) was 24.4 (minimum *C_T_*, 17; maximum *C_T_*, 36) and was <35 in 97.6% of samples. All samples positive by SYBR green-based real-time PCR were successfully identified at the species level by DNA sequencing. This new method should be considered for routine diagnosis of cutaneous leishmaniasis in South America and especially for remote areas, since noninvasive collection tools are easier to use and require fewer precautions for transportation.

## Abstract (precis)

Recent studies have highlighted the interest in noninvasive sampling procedures coupled with real-time PCR methods for the detection of *Leishmania* species in South America. In French Guiana, the sampling method still relied on skin biopsies. Noninvasive protocols should be tested on a large annual cohort to improve routine laboratory diagnosis of cutaneous leishmaniasis. Therefore, we evaluated the performance of a new *Leishmania* detection and species identification protocol involving cotton swabs and SYBR green-based real-time PCR of the Hsp70 gene, coupled with Sanger sequencing.

## INTRODUCTION

Leishmaniases are vector-borne diseases caused by parasites of the genus *Leishmania* (Kinetoplastida: Trypanosomatidae). Cutaneous leishmaniasis (CL) represents an important public health issue in South America, where mucosal and strictly cutaneous forms of the disease can be observed (1). The estimated annual incidence in the Americas ranges from 187,000 to 307,000 new cases (2). In French Guiana, between 100 and 200 cases are reported each year, among a population of roughly 250,000 inhabitants (3, 4). Proper identification of Leishmania braziliensis and L. guyanensis is of paramount importance in French Guiana, where the treatment of the former relies on meglumine antimoniate or amphotericin B, while pentamidine remains the first-line treatment for the latter (4–6). French and European therapeutic guidelines allow the use of topical treatment for small (<4-cm^2^), nonnumerous lesions not involving the head or neck and not originating in Bolivia (7, 8). However, strains of L. braziliensis in French Guiana possess high mucosal tropism, and systemic treatment is proposed to all patients seen with CL (3, 6).

Progress in the diagnosis of leishmaniasis depends on the development of effective methods and the discovery of suitable biomarkers (9). In French Guiana, diagnostic methods for CL have benefited from several breakthroughs in recent years. Conventional PCR-restriction fragment length polymorphism (RFLP) on skin biopsy specimens was introduced in 2007 and helped revise the epidemiology of CL thanks to improved sensitivity of parasite detection (3, 10). Indeed, the sensitivity of PCR-RFLP is superior to that of other parasitological methods such as microscopy or culture, particularly for samples with low parasite density (9). In 2016, matrix-assisted laser desorption ionization–time of flight mass spectrometry (MALDI-TOF MS) was also introduced, allowing *Leishmania* identification from positive cultures through the online reference mass-spectral library MSI (11). But while PCR-RFLP targeting the RNA polymerase II gene has good taxonomic resolution, its power to distinguish between *Leishmania* complexes and species is often insufficient (10). Similarly, MALDI-TOF MS identification requires culture positivity, which is rarely more than 70% efficient (9). A high parasite load (>3 × 10^6^/ml) is also required for species identification (11). Thus, there is a need to develop more-effective and more-sensitive diagnostic methods.

A few recent studies have hinted at the efficiency of new, noninvasive procedures. In a cohort of 55 patients in Brazil, the diagnostic accuracies of swab and biopsy samplings were compared using SYBR green- and TaqMan-based real-time PCR. The authors established the superiority of SYBR green-based PCR and the equal performances of swab and biopsy samplings (12). In Peru, quantification of *Leishmania* DNA showed that the detection of parasite DNA was more efficient with cytology brushes than with dermal scrapings or skin biopsies (13). Several other studies in South America reported the superiority of swabs over aspiration (14) and the high sensitivity of cytology brushes for mucosal (15) or cutaneous (16) lesions. A small study in Brazil also confirmed that results with swabs were superior to those with biopsies (17). In French Guiana, sampling techniques have not been updated, and the diagnosis of CL has still relied on skin biopsies. This has given rise to technical and logistical issues, particularly in the Health Centres for Remote Areas, which offer primary care to the Amerindian and Maroon populations of the rainforest regions (18). These issues have led to a rethinking of the sampling technique in an effort to simplify the diagnosis of CL, notably in the remote areas, where its incidence is significant (18, 19).

The aim of this study was to validate the use of cotton swabs coupled with SYBR green-based real-time PCR of the Hsp70 gene as a new tool for the diagnosis of cutaneous leishmaniasis on the large annual cohort in French Guiana. This new protocol also includes the assessment of DNA sequencing of the *Leishmania* target gene *HSP70*, which has been recommended for *Leishmania* typing (20).

## MATERIALS AND METHODS

### Study design and ethics.

**FIG 1:**
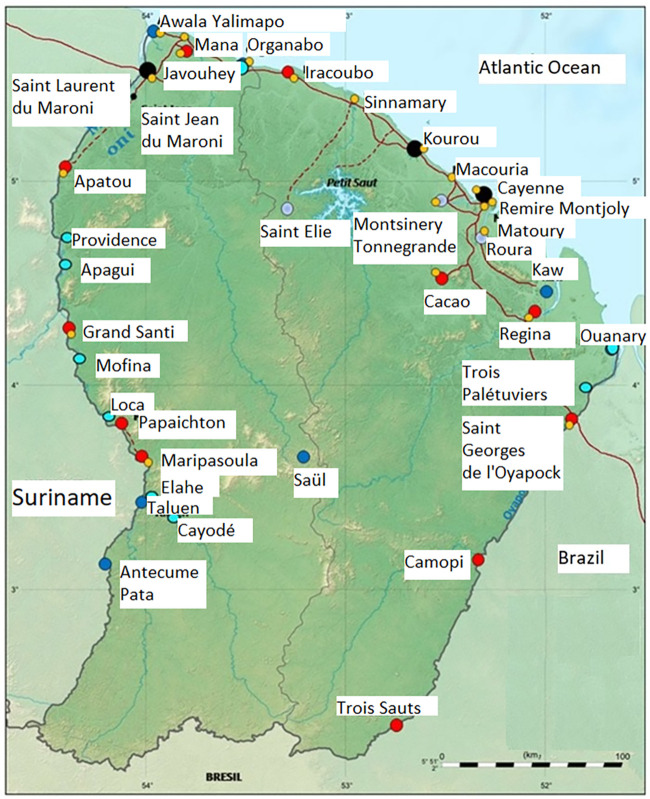
Map of French Guiana and the Health Centres for Remote Areas, showing hospital centers (black dots), remote health centers with a permanent physician (red dots), health centers with a permanent nurse but no physician (blue dots), closed health centers (gray dots), pregnancy centers (yellow dots), and temporary health missions (sky-blue dots).

The study was conducted during a 1-year follow-up, between May 2017 and May 2018, in the Cayenne General Hospital and in the Health Centres for Remote Areas. These 16 centers offer primary care in the remote locations of the hinterland, mostly inhabited by Amerindian and Maroon populations (Fig. 1). Patients with skin lesions compatible with cutaneous leishmaniasis who were sampled during routine clinical care were included. Patients with only nonulcerated lesions (nodules, papules, lymphangitis) were sampled, but their data were not included in the analysis. Indeed, for these patients, sampling with a cotton swab can be performed only in the hole left by the skin biopsy and thus cannot replace this technique.

If necessary, clinical expertise was provided by a dermatologist at the general practitioner’s request, either in outpatient consultation, by teledermatology, or by hospitalization. For each patient, the following samples were obtained: three smear slides of scrapings for microscopic examination; two 4-mm punch biopsy specimens for parasite culture and PCR-RFLP, respectively; and two swab samples for real-time PCR (Dry Polyester swabs, 159C; Copan, Brescia, Italy). Clinical and epidemiological characteristics were also recorded for all suspect cases.

This study was part of the research work of the French National Reference Centre for Cutaneous Leishmaniasis, of which the Cayenne Hospital is an associated laboratory. The identification of *Leishmania* species corresponded to the surveillance and alert mission of the National Reference Centre (CNR) of *Leishmania*. The conduct of the study complied with the French rules for research involving human subjects and the Helsinki Declaration guidelines. All patients were informed (through leaflets and posters in several local languages) that data and analysis results might be used in research and scientific publications and that they had a right to refuse. Informed consent was recorded in medical files. Under French law, no further legal clearance was required.

The STARD guidelines (21) were followed, including blinding of index and reference diagnostic tests. Clinical information was not available to the performers of diagnostic tests. Smear and culture, conventional PCR-RFLP, and real-time PCR were performed by three different teams with no access to any other team’s results.

### Sampling procedure.

**FIG 2:**
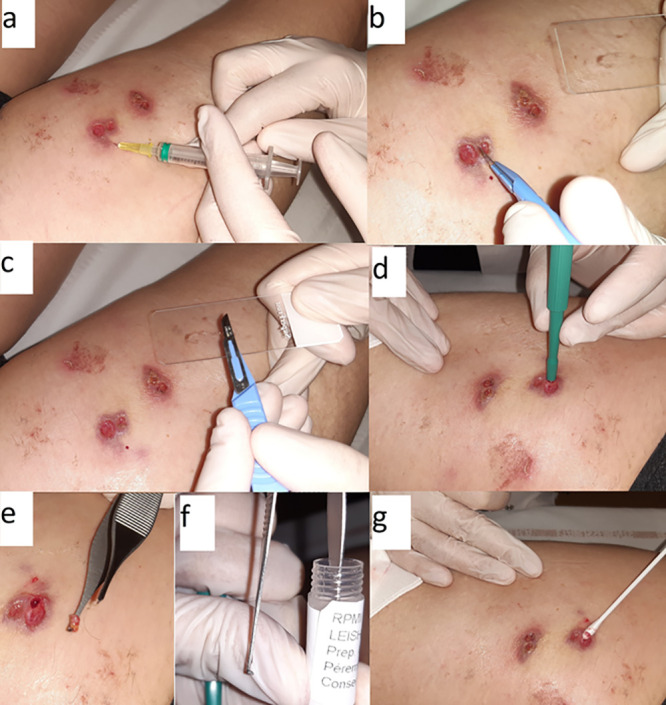
Pictures of different sampling procedures (smear, skin biopsy, cotton swab) used for the diagnosis of cutaneous leishmaniasis, French Guiana, 2017–2018. (a) Lidocaine injection for anesthesia; (b) scraping of the inner border with a scalpel; (c) smearing with the scalpel on a microscope slide; (d) skin biopsy with a 4-mm punch; (e) removal of the skin plug from the biopsy punch; (f) storage of the plug sample in a tube filled with RPMI medium; (g) gentle pressing, with rotation, of a cotton swab against the lesion center.

Prior to any sampling, ulcerated lesions were cleaned with physiological serum and the surrounding areas were disinfected with 70% ethanol or another antiseptic in order to avoid contamination. Cotton swabs were gently pressed and rotated 360° at the center of the ulcerated lesion, after removal of the crust if necessary. Skin biopsy and swab samples were taken at the centers of the lesions in order to retrieve higher parasitic loads (13). Skin biopsy specimens were placed in RPMI medium (RPMI 1640; Sigma, St. Louis, MO) supplemented with 20% fetal calf serum, 1% nonessential amino acids, and 50 IU/ml penicillin, stored at −4°C, and refrigerated using cold packs during transport (14). Smears and swab samples were stored and sent at ambient temperature. Swabs were kept in dry transport tubes. All analyses were then performed at the Parasitology-Mycology Laboratory of the Cayenne Hospital. Pictures depicting the different sampling methods are presented in Fig. 2.

### DNA extraction and *Leishmania* amplification.

DNA was extracted with the QIAamp DNA minikit (Qiagen, Hilden, Germany) according to the manufacturer’s protocol for cotton swabs and biopsies. *Leishmania* amplification and species identification by PCR-RFLP (targeting the RNA polymerase II gene) from skin biopsy specimens were performed according to routine laboratory protocol (10). *Leishmania* amplification by SYBR green-based real-time PCR on cotton swabs was performed using a 234-bp fragment of the *hsp70* gene designed for the diagnosis of American CL (subgenus *Viannia*) using previously published primers (5′-GGA CGA GAT CGA GCG CAT GGT-3′ and 5′-TCC TTC GAC GCC TCC TGG TTG-3′) (22). Reactions were conducted in a total volume of 25 μl, containing 5 μl of each DNA sample, 12.5 μl of SYBR green 2× Master Mix (Applied Biosystems), 0.5 μl of each primer (final concentration set at 2.5 μM), and 6.5 μl of H_2_O. Negative and positive controls were included in each DNA amplification run. Negative controls consisted of ultrapure water. The positive control was a DNA extract from a culture of reference strain Leishmania guyanensis MHOM/GF/97/LBC6. Real-time PCR was performed on a thermocycler (Applied Biosystems 7500) as follows: a holding stage at 50°C for 2 min, followed by 95°C for 10 min; a cycling stage at 94°C for 30 s, followed by 63°C for 1 min (40 cycles); and finally, a melt curve stage at 95°C for 15 s, followed by 60°C for 1 min. Amplified products obtained in the HSP70 targeting were sent for Sanger sequencing to Eurofins Genomics, Les Ulis/Courtaboeuf, France. Sequences were made available on a secured server within a week and were compared with reference strains found in the GenBank database using BLAST (https://blast.ncbi.nlm.nih.gov/Blast.cgi).

### *Leishmania* culture.

*Leishmania* cultures were performed according to routine laboratory protocol. Briefly, biopsy specimens were cultivated in RPMI medium (RPMI 1640; Sigma, St. Louis, MO) supplemented with 20% fetal calf serum, 1% nonessential amino acids, and 50 IU/ml penicillin for as long as 21 days at 28°C and were observed under a microscope at 3-day intervals (10). *Leishmania* species were identified by MALDI-TOF MS from positive cultures using the online identification platform MSI, as previously published (11).

### Statistical analysis.

Univariate analysis was used to analyze each factor of interest between patients with positive or negative laboratory tests. Odds ratios (ORs) were estimated with 95% confidence intervals (CI). Associations were deemed significant with a *P* value of <0.05. All analyses were conducted using Stata software (StataCorp, College Station, TX, USA).

## RESULTS

**FIG 3:**
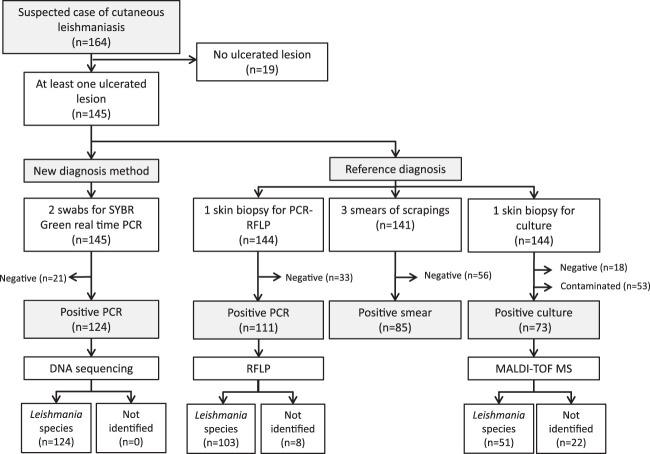
Flow chart of the study population and positive tests in the annual cohort of patients with suspected cutaneous leishmaniasis, French Guiana, 2017–2018.

During the study period, 164 patients were seen with suspected cutaneous leishmaniasis. Nineteen of them were excluded from analysis, since they presented only nonulcerated lesions (nodules, papules, or lymphangitis). A total of 145 patients with at least one ulcerated lesion formed the study population. Among them, 19 (13.1%) were negative by all tests and 126 (86.9%) had at least one positive test (Fig. 3). Smear sensitivity was 68% overall but differed between samples shipped from the remote health centers (62%) and those from the Cayenne Hospital (80%) (Table 1). The overall sensitivity of culture was lower than that of smears (58%), due to a high rate of contamination by either fungi or bacteria, accounting for 36.8% (53/144) of skin biopsy cultures and 78.8% (41/52) of false-negative samples. However, the samples from remote areas accounted for 75.5% (40/53) of the contaminated cultures. Thus, the sensitivity of cultures from remote areas was much lower (49%) than that in Cayenne (76%). Skin biopsy specimens from Cayenne had a significantly higher chance of being positive than those from remote areas (OR, 2.18 [95% CI, 1.02 to 4.67]; *P* = 0.0278).

**TABLE 1:** Comparison of performances of four diagnostic tests for 145 patients with suspected cutaneous leishmaniasis, French Guiana, 2017 to 2018

Diagnostic test and region	No. of samples				Sensitivity (%)	OR (95% CI)	*P* value
	True positive	True negative^*a*^	False negative^*a*^	Not done			
Smear	85	16	40	4	**68**		
Cayenne	33	8	8	2	80	1.48 (0.69–3.20)	0.2731
Remote areas	52	8	32	2	62	1	
Culture on skin biopsy specimen	73	19 (12)	52 (41)	1	**58**		
Cayenne	32	9 (4)	10 (9)	0	76	**2.18 (1.02–4.67)**	**0.0278**
Remote areas	41	10 (8)	42 (32)	1	49	1	
PCR-RFLP on skin biopsy specimen	111	19	14	1	**89**		
Cayenne	39	9	2	1	95	0.99 (0.42–2.45)	0.9864
Remote areas	72	10	12	0	86	1	
SYBR green-based PCR on swab	124	19	2	0	**98**		
Cayenne	41	9	1	0	98	0.54 (0.19–1.56)	0.1965
Remote areas	83	10	1	0	99	1	

a The number of contaminated cultures is given in parentheses.

Among the 53 contaminated cultures, most were due to yeasts. Indeed, we report 50.9% (27/53) yeasts, 22.6% (12/53) bacteria, 18.9% (10/53) filamentous fungi, and 7.5% (4/53) associations of yeasts and filamentous fungi. Among the 73 *Leishmania*-positive cultures, mass spectrometry identification of species was achievable for 51 isolates, while 22 isolates could not be identified because of insufficient parasite density (<3 × 10^6^/ml) (11). PCR tests yielded the best performance. Real-time SYBR green-based PCR on swabs was more sensitive (98%) than PCR-RFLP on skin biopsy specimens (89%). The performance of SYBR green-based PCR was similar for samples from Cayenne (sensitivity, 98%) and those from the remote health centers (99%). The mean PCR cycle threshold (*C_T_*) for SYBR green-based PCR was 24.4 (minimum, 17; maximum, 36), and the *C_T_* was <35 in 97.6% of positive samples.

In 13 cases, SYBR green-based PCR was positive on swab samples while PCR-RFLP was negative on skin biopsy specimens. These conflicting swab samples were also amplified with PCR-RFLP: in 12 cases, PCR was negative again. In only one case, PCR-RFLP on a swab extract gave a positive result. By SYBR green-based PCR, the mean *C_T_* for these samples was high (31.4). For these patients, smear and culture were also negative. On the other hand, only two false-negative results were recorded with SYBR green-based PCR: the diagnosis was made with a positive culture in one case and with positive PCR-RFLP in the other.

All samples positive by SYBR green-based real-time PCR allowed species identification. In seven cases, RNA polymerase II (PCR-RFLP) allowed identification only at the genus level (*Leishmania* spp.), while Hsp70 sequencing (SYBR green-based PCR) allowed the identification of L. guyanensis (*n* = 4), L. braziliensis (*n* = 2), and L. lainsoni (*n* = 1). For two patients, the species identified were discordant between the two targeted sequences. PCR-RFLP identified L. braziliensis and L. guyanensis, while Hsp70 sequencing identified L. naiffi and L. braziliensis, respectively.

The clinical and epidemiological characteristics of the 145 patients with compatible lesions were compared for those with positive or negative tests (Table 2). A predominance of males and adults was observed in both the positive (69.0% and 89.7%) and negative (68.4% and 89.5%) groups. Lesions on the lower limbs were found in 53.2% of patients with proven CL. There was no statistical difference in clinical characteristics between the two groups. The median number of lesions (one) was similar in the two groups. Among the 126 patients with at least one positive test, four different *Leishmania* species were isolated: L. guyanensis (105 patients [83.3%]), L. braziliensis (16 patients [12.7%]), L. naiffi (2 patients [1.6%]), and L. lainsoni (1 patient [0.8%]). In two cases, the species remained unidentified.

**TABLE 2:** Clinical and epidemiological characteristics of 145 patients testing positive or negative for cutaneous leishmaniasis, French Guiana, 2017–2018

Characteristic	No. (%) of patients with the indicated characteristic and the following diagnosis:		OR (95% CI)	*P* value
	Positive (*n* = 126)	Negative (*n* = 19)		
Age^*a*^				
Adults	113 (89.7)	17 (89.5%)	1	
Children (<18 yr)	13 (10.3)	2 (10.5)	0.98 (0.19–9.68)	0.98
Gender				
Male	87 (69.0)	13 (68.4)	1	
Female	39 (31)	6 (31.6)	0.97 (0.32–3.35)	0.95
Origin				
Cayenne	42 (33.3)	9 (47.4)	1	
Health centers	84 (66.7)	10 (52.6)	1.8 (0.59–5.33)	0.23
Localization^*b*^,^*c*^				
Head	12 (9.5)	0 (0)	2.04 (0.27–91.86)	0.49
Nonhead	106 (84.1)	19 (100)	1	
Lower limbs	67 (53.2)	12 (63.2)		
Trunk	21 (16.6)	2 (10.5)		
Upper limbs	49 (38.8)	9 (47.4)		
Type of lesion^*b*^				
Ulceration only	120 (95.2)	18 (94.7)	1	
Ulceration plus another type	6 (4.8)	1 (5.3)	0.9 (0.01–43.66)	0.92
Nodule	2 (1.6)	1 (5.3)		
Papules	2 (1.6)	0		
Lymphangitis	3 (2.4)	0		

a The median ages were 33 years for positive patients and 45 years for negative patients.

b Some patients had several types and several different localizations of lesions.

c Localization was missing for eight patients.

## DISCUSSION

This study validates the use of swab sampling coupled with real-time SYBR green-based PCR and Hsp70 sequencing for the diagnosis of CL in French Guiana. The large number of patients included in this cohort and the utilization of this technique in the real-time routine situation of the rainy season in French Guiana confirms that swab sampling offers an interesting alternative to skin biopsy in the field. Another strength of this study is its multicentric design, since patients were included not only in the referral hospital but also in 16 remote health centers, confirming the validity of these data for isolated populations of the rainforest area.

Since routine diagnosis of cutaneous leishmaniasis often relies on several different tests with limited sensitivities, we considered all patients with at least one positive test to have confirmed diagnoses. We showed good agreement between the results of real-time SYBR green-based PCR on swab samples and PCR-RFLP on skin biopsy specimens. However, real-time PCR on swabs showed the highest sensitivity (98%). Thirteen samples with high *C_T_* values (mean, 31.4) were positive only by SYBR green-based PCR. The fact that we tried to amplify these swab extracts with PCR-RFLP and still obtained negative results suggests a lower sensitivity of this PCR method in cases of low parasite densities rather than an extraction or amplification failure. Thus, the improved sensitivity of SYBR green-based real-time PCR was probably due not only to the sampling technique but also to the PCR method. It has been shown that higher parasite loads are retrieved from the upper skin layers by using superficial samplings, such as brushes or scrapings, than by biopsies reaching the deep skin layers (13, 23). Therefore, increasing the ratio of parasitic DNA to human DNA with a noninvasive sampling technique allows noninvasive procedures to outperform skin biopsies. In our study, the mean *C_T_* for SYBR green-based real-time PCR was very low (*C_T_*, 24), and most samples (97.6%) were positive with a *C_T_* of <35. This result underlines the important parasite load yielded by swab sampling.

SYBR green-based real-time PCR of Hsp70 offered better performance than PCR-RFLP of RNA polymerase II. The latter was introduced in French Guiana in 2010 (10) and became the reference method for species identification (3). This technique presented several advantages, since it was available in the Cayenne Hospital and did not require sending samples to mainland France. It also allowed proper identification of most samples of L. guyanensis and L. braziliensis, which is the first and foremost need for clinicians in this territory. However, the authors acknowledged the limitations of this PCR for the diagnosis of other species (10). Moreover, since PCR-RFLP relies on the detection of amplicons by electrophoresis, interpretation of species-specific bands might be operator dependent. Using Hsp70 as a target for PCR probably helped improve the sensitivity and accuracy of diagnosis. Previous studies have shown that Hsp70 offers the best *Leishmania* typing tool, particularly in New World CL (20). Our study confirms these findings in the large annual cohort of patients with CL in French Guiana, where HSP70 sequencing allowed good discrimination between species, notably between L. guyanensis and L. braziliensis. However, we used a short (234-bp) sequence, which could potentially lead to misdiagnosis, since the accuracy of DNA sequencing identification is known to vary importantly according to the Hsp70 primers used (20). The use of a larger sequence would certainly improve the accuracy of species identification and phylogenetic analysis. It is unclear whether Hsp70 can successfully distinguish L. guyanensis from L. panamensis, but this distinction has limited clinical importance, due to the high efficacy of pentamidine for both species (8). On the other hand, a large (1,286-bp) Hsp70 sequence was one of the best markers for discrimination between L. braziliensis and L. peruviana, an important distinction for other areas of South America, where both species are present (24). Hsp70 as a target gene is also likely to provide more-accurate epidemiological data on rare species such as L. naiffi or L. lainsoni. Few cases of infections caused by these species had been reported by the time PCR-RFLP was introduced in French Guiana. However, new cases have been reported since 2010 (25), and precise surveillance of these species should be allowed by routine diagnosis tools.

Concerning other diagnostic methods, the sensitivity of smears on scrapings was unexpectedly high (68%). Other studies reported lower smear positivity rates of 29.7% (12), 14.3% (14), and 43.4% (16). This is an interesting finding, since the sensitivity of smears is highly dependent on the number and dispersion of parasites but also on the sampling process and technical skills (9). In French Guiana, active training for smear preparation is provided to all health providers. This could explain the quality of sampling, particularly in samples collected from the Dermatology Department (sensitivity, 80%). However, staff training in the remote health centers should be improved to reach the level of sampling quality observed in the Cayenne Hospital. Indeed, in areas of endemicity, smears remain the quickest and cheapest way of achieving a diagnosis of CL, although they do not provide species identification (9).

Parasite culture on skin biopsy specimens was less sensitive than smears in this study (58%), perhaps due to the high rate of contamination by fungi or bacteria (36.8%). Contamination was particularly frequent for biopsy specimens originating from the Health Centres for Remote Areas, which represented 75% of contaminated cultures. This high proportion of contaminated cultures could be explained by the longer transportation time or a broken cold chain during transport. Many of these samples are shipped from the remote areas to Cayenne by small aircraft or canoes. While culture on skin biopsy specimens is useful for growing large amounts of parasite strains for research purposes, its utility for routine diagnosis in remote equatorial areas seems weak. According to some studies (26), using 5-fluorocytosine could have prevented yeast contamination, which accounted for the majority of contaminated cultures. However, empirical experience from both our laboratory and the National Reference Centre of Montpellier indicates that antifungals might hamper *Leishmania* growth. These empirical findings are also supported by some publications (27). Transport conditions probably play an important part in preventing contamination. Thus, biopsy specimens from the Cayenne Hospital benefited from a short transport time and were more likely to be positive (*P* = 0.0278).

Clinically, we did not find any significant difference between patients with positive and negative tests. In French Guiana, the clinical features of CL (characteristic ulcerative lesions on the limbs) are well known to most clinicians (4). General practitioners can rely on easy access to dermatologists through teledermatology or outpatient consultation (18). This system could explain the high proportion of positive diagnoses (86.9% of suspect patients had at least one positive test). Most patients with negative tests presented staphylococcal ecthyma or vascular ulcers.

The introduction of noninvasive sampling procedures allows painless, simpler sample collection for patients and relieves health professionals of cumbersome logistical issues. Conversely, there was no difference in terms of efficiency between the collection of swab samples for SYBR green-based PCR in the main hospital and that in the remote health centers. Logistical issues hampering the transport of biopsy specimens from these remote areas do not seem to apply to cotton swabs. Indeed, noninvasive collection tools are easier to use and require fewer precautions for transportation (14, 15). These results are promising for other settings in South America, but a systematic comparison of different transport temperatures would provide even more-solid data. As suggested by other authors for cytology brushes (15), swabs can be transported from different remote settlements and gathered in a reference center for DNA amplification before amplicons are sent to a sequencing platform. In our setting, the mean time to diagnosis with SYBR green-based real-time PCR is now 2 weeks, and sequencing costs only €4.0 per sequence.

Since the completion of this study, the performance of skin biopsies has been stopped for patients seen with ulcerated lesions in the health centers for remote areas. Biopsies remain necessary for patients with only nonulcerated lesions, such as papules and nodules. However, our routine protocol in French Guiana now includes sampling with cotton swabs in the hole left by the biopsy. Indeed, although we did not present these results in our analyses, SYBR green-based PCR was also performed on swab samples taken from the biopsy holes of patients seen during the study period with papules and nodules, and this procedure yielded very good results. This supplementary technique can improve the overall sensitivity of sampling in cases of negative or contaminated biopsy specimens.

This large study in the real-life situation of a territory where CL is endemic confirms the utility of this noninvasive technique along with the development of effective detection methods such as real-time SYBR green-based PCR coupled with DNA sequencing. In the future, the introduction of a local sequencing platform could reduce the time to diagnosis and allow faster treatment for patients with painful lesions.
